# Stevioside Attenuates Insulin Resistance in Skeletal Muscle by Facilitating IR/IRS-1/Akt/GLUT 4 Signaling Pathways: An In Vivo and In Silico Approach

**DOI:** 10.3390/molecules26247689

**Published:** 2021-12-20

**Authors:** Abilasha Deenadayalan, Vijayalakshmi Subramanian, Vijayalakshmi Paramasivan, Vishnu Priya Veeraraghavan, Gayathri Rengasamy, Janaki Coiambatore Sadagopan, Ponnulakshmi Rajagopal, Selvaraj Jayaraman

**Affiliations:** 1 Department of Anatomy, Saveetha Institute of Medical & Technical Sciences, Chennai 602 105, India; abipriya.papu@gmail.com (A.D.); svlgp65@gmail.com (V.S.); 2 Department of Anatomy, Asan Memorial Dental College and Hospitals, Asan Nagar, Chengalpattu, Chennai 602 105, India; 3 Department of Pharmacology, Asan Memorial Dental College and Hospitals, Asan Nagar, Chengalpattu, Chennai 602 105, India; vijayalakshmi.paramasivan@gmail.com; 4 Department of Biochemistry, Saveetha Dental College & Hospital, Saveetha Institute of Medical & Technical Sciences, Chennai 600 077, India; vishnupriya@saveetha.com (V.P.V.); gayathri.sdc@saveetha.com (G.R.); 5 Department of Anatomy, Bhaarat Medical College, Selaiyur, Chennai 600 073, India; janaki098@gmail.com; 6 Department of Central Research Laboratory, Meenakshi Ammal Dental College and Hospitals, Chennai 600 095, India; drponnulakshmi.researchscientist@madch.edu.in

**Keywords:** type-2 diabetes, insulin resistance, Stevioside, gastrocnemius muscle, oxidative stress, antioxidants, insulin signaling

## Abstract

Type-2 diabetes mellitus (T2DM), the leading global health burden of this century majorly develops due to obesity and hyperglycemia-induced oxidative stress in skeletal muscles. Hence, developing novel drugs that ameliorate these pathological events is an immediate priority. The study was designed to analyze the possible role of Stevioside, a characteristic sugar from leaves of *Stevia rebaudiana* (Bertoni) on insulin signaling molecules in gastrocnemius muscle of obesity and hyperglycemia-induced T2DM rats. Adult male Wistar rats rendered diabetic by administration of high fat diet (HFD) and sucrose for 60 days were orally administered with SIT (20 mg/kg/day) for 45 days. Various parameters were estimated including fasting blood glucose (FBG), serum lipid profile, oxidative stress markers, antioxidant enzymes and expression of insulin signaling molecules in diabetic gastrocnemius muscle. Stevioside treatment improved glucose and insulin tolerances in diabetic rats and restored their elevated levels of FBG, serum insulin and lipid profile to normalcy. In diabetic gastrocnemius muscles, Setvioside normalized the altered levels of lipid peroxidase (LPO), hydrogen peroxide (H_2_O_2_) and hydroxyl radical (OH*), antioxidant enzymes (CAT, SOD, GPx and GSH) and molecules of insulin signaling including insulin receptor (IR), insulin receptor substrate-1 (IRS-1) and Akt mRNA levels. Furthermore, Stevioside enhanced glucose uptake (GU) and oxidation in diabetic muscles by augmenting glucose transporter 4 (GLUT 4) synthesis very effectively in a similar way to metformin. Results of molecular docking analysis evidenced the higher binding affinity with IRS-1 and GLUT 4. Stevioside effectively inhibits oxidative stress and promotes glucose uptake in diabetic gastrocnemius muscles by activating IR/IRS-1/Akt/GLUT 4 pathway. The results of the in silico investigation matched those of the in vivo study. Hence, Stevioside could be considered as a promising phytomedicine to treat T2DM.

## 1. Introduction

The Diabetes Mellitus Association predicted that 578 million people will have diabetes in 2030 and the number will increase by 51% (700 million) in 2045 [1]. It is characterized by absolute or relative deficiencies in insulin secretion (Type 1 diabetes) and/or insulin action (Type 2 diabetes) associated with chronic hyperglycemia and disturbances of carbohydrate, lipid and protein metabolism [2]. Diet plays a key role in induction of diabetes in humans [3]. Increased high fat diet intake and Western diets have been linked to insulin resistance to be associated with high risk of diabetes mellitus and related metabolic diseases [4,5]. To maintain a constant blood glucose level, the body relies on two hormones produced in the pancreas that have opposite actions: insulin and glucagon [6]. Over the years, it has become more and more evident that the development of type 2 diabetes mellitus (T2DM) is fueled by bad diets and unhealthy lifestyles which majorly contain high fat or the sugar [7]. Nutritional overload in the form of high dietary intake of fats and non-glycolysis sugar (sucrose) plays a major role in the accelerating gluconeogenesis and inhibited glycogenolysis [8]. High-fat (HFD) and high-sucrose diets (HSD) reduce insulin suppression of glucose production in vivo, alters gluconeogenesis by increase glucose-6-phosphatase (G-6-Pase) activity in whole cell homogenates [9]. Podolin et al. [10] reported that high-fat diet feeding increased fat mass or obesity, the absolute rate of lipolysis and the contribution of estimated nonglycerol gluconeogenesis to hyperglycemia. These diabetic environments trigger oxidative stress, resulting from the production of free radicals via glucose auto-oxidation, increase ROS and autophagy and have a great role in the initiation and progression of diabetic complications [11]. To avoid the complications and alternation in diet-induced glucose metabolism, it is important to maintain normal gluconeogenic rates, possible through studying the molecular mechanisms regulating hepatic gluconeogenesis. The modern lifestyle, genetics and nutritional overload [12] attributed to prevalence and outcomes of T2D with various complications develop including both macro and micro-vascular dysfunctions [13,14].

Nowadays, there is a great interest in using medicinal plants and their bioactive compounds for treatment of diabetes. *Steviare baudiana* (*S. baudiana*) is also called sweet herb, sweet leaf, honey leaf, candy leaf and honey yerba [15]. From the past few decades, the study of structural, chemical and functional aspects of *S. rebaudiana* has been in the limelight in order to discover different diterpene glycosides (DGs). Extensive research has led to the identification of more than 30 DGs from *S. rebaudiana* [16]. Among the identified DGs, Stevioside is generally said to have a somewhat unpleasant taste, is non-fermentable and had low caloric value [17]. However, Stevioside is 300-fold sweeter, with a stretched-out timeframe of realistic usability, when contrasted with ordinary sugar [18]. Because of its non-caloric nature, Stevioside, a diterpenoid glycoside comprising an aglycone (steviol) and three particles of glucose, has been widely used as an replacement for hyperglycemic food around the world, including by the European Association and the Assembled States [19]. Because there are no comparable chemicals in individuals, Stevioside and other sweet steviol glycosides, such as steviobioside, rebaudioside (A–F) and ducoside A, are not retained and debased in the digestive system; yet gastrointestinal bacterial verdure can change Steviosides into the metabolites steviol and glucose. The subsequent glucose is devoured by microscopic organisms in the colon and is not assimilated into fundamental dissemination [20]. It has been reported that steviol glycosides exhibit many therapeutic benefits, such as hypoglycemic, anti-hypertensive and anti-inflammatory activities [21]. Studies have shown that stevioside enhances insulin secretion which regulates glucose metabolism, leading to a reduction of blood pressure, and acts as an anti-hyperglycemic agent [22]. The current study was designed to provide an experimental evidence of the effectiveness of Stevioside on the expression of insulin signaling molecules and glucose oxidation in gastrocnemius muscle of type-2 diabetic rats.

## 2. Results

### 2.1. Stevioside Reduced FBG and Improved OGTT in T2DM Rats

**Figure 1:**
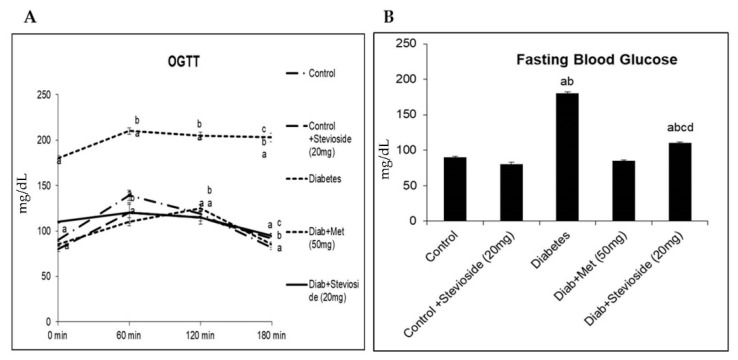
(**A**,**B**) Effect of Stevioside on FBG and OGT in type-2 diabetic rats. Each bar represents mean ± SEM (n = 6) analyzed by one-way ANOVA with Student–Newman–Keul’s multiple comparison test. Significance at *p* < 0.05. ^a^ Significantly different from control group. ^b^ Significantly different from control rats treated with 20 mg Stevioside group. ^c^ Significantly different from diabetic group. ^d^ Significantly different from metformin treated diabetic group.

To evaluate the anti-diabetic potential of Stevioside, FBG levels were measured and OGTT was performed in control and experimental animals. As shown in Figure 1, Stevioside treatment reduced the elevated FBG levels in diabetic rats as effectively as metformin. After glucose load, group III diabetic rats displayed a gradual increase in blood glucose level which reached its maximum value at 1 h. This elevated value did not reach the normal range even after two hours post glucose load, which is indictive of glucose intolerance. However, treatment with Stevioside improved the glucose tolerance in diabetic rats as effectively as the conventional anti-diabetic drug metformin (Figure 1A,B).

### 2.2. Effect of Stevioside on Fasting Serum Insulin T2DM Rats

**Figure 2:**
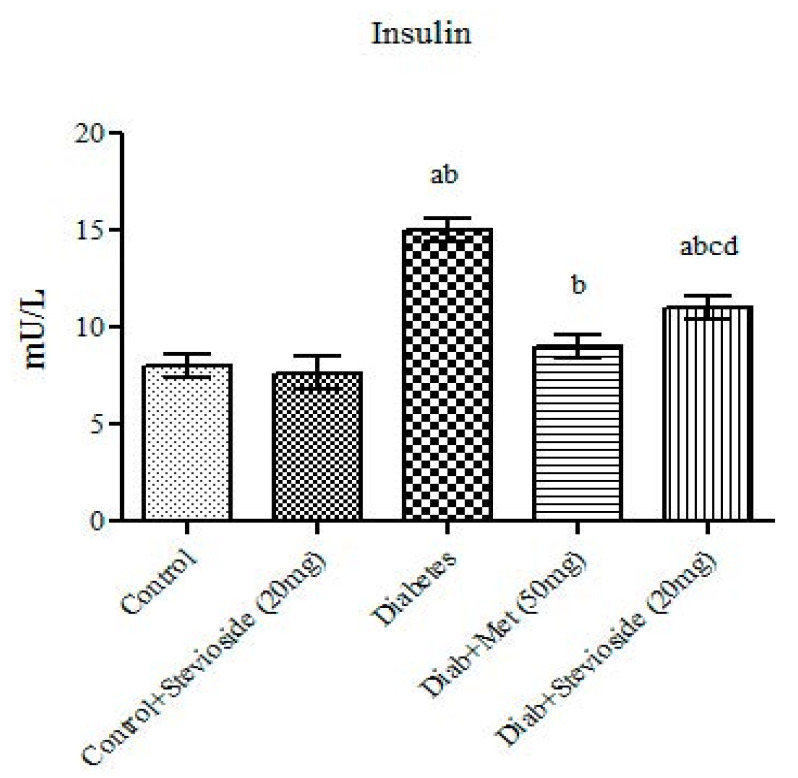
Effect of Stevioside on serum insulin in type-2 diabetic rats. Each bar represents mean ± SEM (n = 6) analyzed by one-way ANOVA with Student–Newman–Keul’s multiple comparison test. Significance at *p* < 0.05. ^a^ Significantly different from control group. ^b^ Significantly different from control rats treated with 20 mg Stevioside group. ^c^ Significantly different from diabetic group. ^d^ Significantly different from metformin treated diabetic group.

Figure 2 represents the level of serum insulin in the different groups of rats under study. Group III rats manifest features of severe insulin resistance which is evidenced by the elevations in the fasting serum insulin levels. However, treatment with Stevioside restored these parameters to near normal range thereby proving its insulin sensitizing potential.

### 2.3. Effect of Stevioside on Liver and Kidney Function Markers in Diabetic Rats

**Figure 3:**
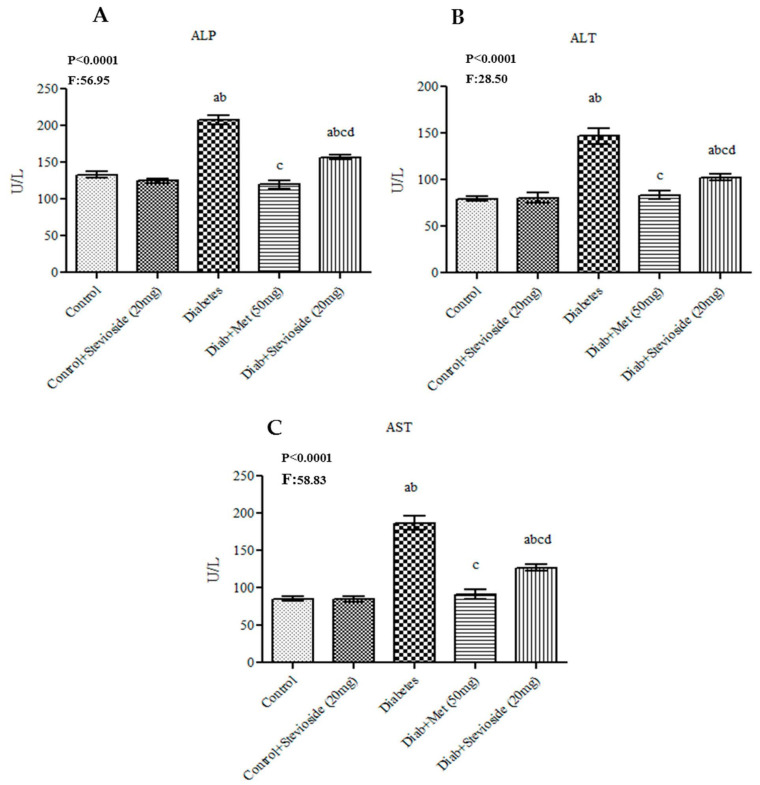

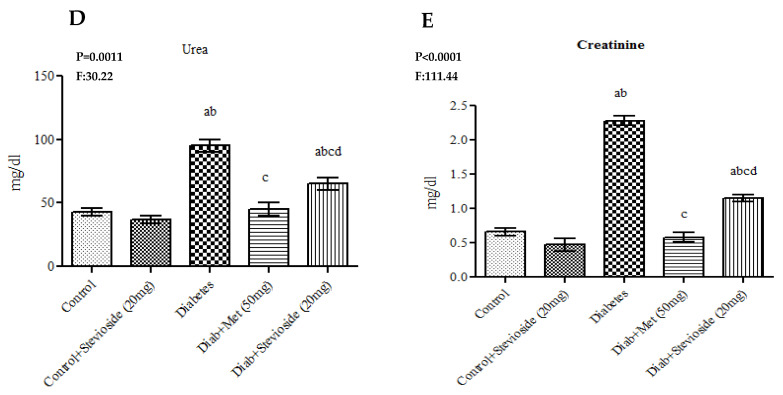
(**A**–**E**) Effect of Stevioside on liver and kidney function markers in type-2 diabetic rats. Each bar represents mean ± SEM (n = 6) analyzed by one-way ANOVA with Student–Newman–Keul’s multiple comparison test. Significance at *p* < 0.05. ^a^ Significantly different from control group. ^b^ Significantly different from control rats treated with 20 mg Stevioside group. ^c^ Significantly different from diabetic group. ^d^ Significantly different from metformin treated diabetic group.

In group III diabetic animals, liver function markers like ALT, AST and ALP (Figure 3A–C) and renal function markers like urea and creatinine (Figure 3D,E) were observed to be high. Stevioside lowered it as effectively as the standard drug metformin.

### 2.4. Stevioside Effect on Serum Lipid Profile

**Figure 4:**
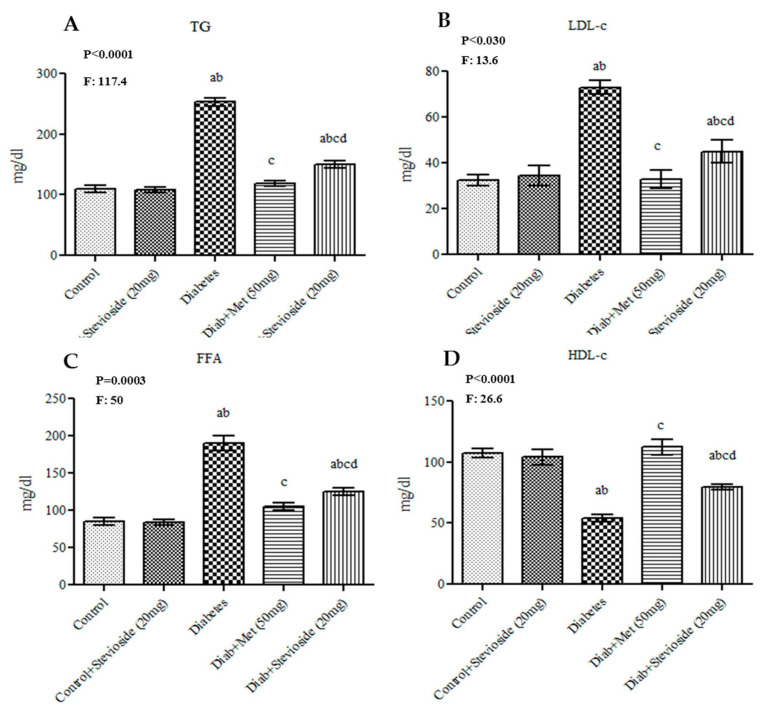
(**A**–**D**): Effect of Stevioside on lipid markers in type-2 diabetic rats. Each bar represents mean ± SEM (n = 6) analyzed by one-way ANOVA with Student–Newman–Keul’s multiple comparison test. Significance at *p* < 0.05. ^a^ Significantly different from control group. ^b^ Significantly different from control rats treated with 20 mg Stevioside group. ^c^ Significantly different from diabetic group. ^d^ Significantly different from metformin treated diabetic group.

To appraise the hypolidemic properties of Stevioside, the serum levels of TG, FFA, HDL-c and LDL-c were estimated in various treatment groups (Figure 4A–D). Diabetic rats manifested features of dyslipidemia which is evident by the significant increase in their serum levels of TG, FFA and LDL-c and low levels of HDL when compared with control groups. However, Stevioside alleviated dyslipidemia in diabetic rats as effectively as metformin by restoring these lipid profile values to normalcy as illustrated in Figure 4A–D.

### 2.5. Stevioside Effect on Oxidative Stress Markers in the Gastrocnemius Muscle

**Figure 5:**
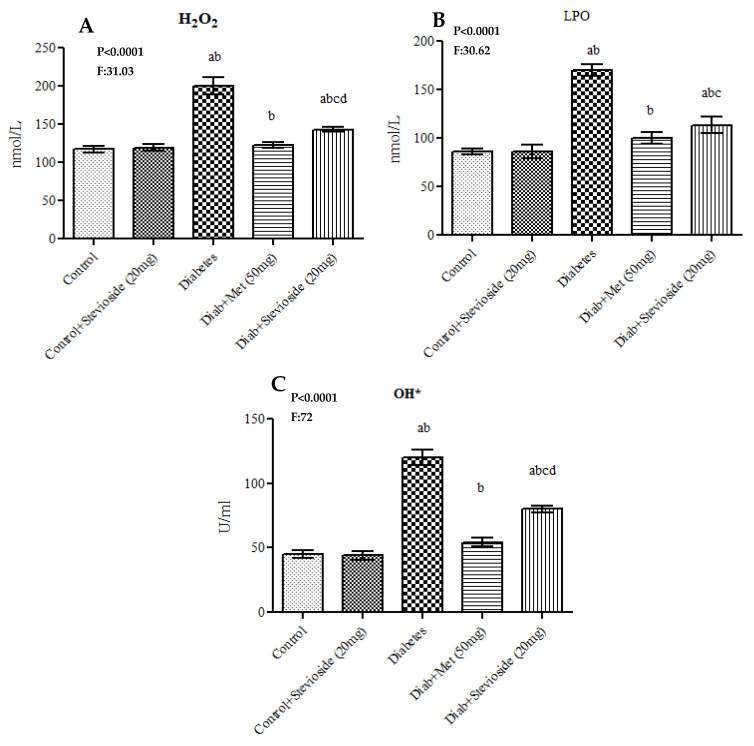
(**A**–**C**) Effect of Stevioside on LPO, H_2_O_2_ and OH* in gastrocnemius muscle of type-2 diabetic rats. Each bar represents mean ± SEM (n = 6) analyzed by one-way ANOVA with Student–Newman–Keul’s multiple comparison test. Significance at *p* < 0.05. ^a^ Significantly different from control group. ^b^ Significantly different from control rats treated with 20 mg Stevioside group. ^c^ Significantly different from diabetic group. ^d^ Significantly different from metformin treated diabetic group.

HFD and sucrose-induced T2DM rats showed a substantial rise in the levels of oxidative stress markers including H_2_O_2_, °OH and LPO in the gastrocnemius muscles compared to control groups, whereas Stevioside treatment remarkably reduced the elevations in these oxidative stress markers in diabetic rats suggestive of its ROS scavenging potentials (Figure 5).

### 2.6. Stevioside Elicits the Levels of Antioxidant Enzymes in the Gastrocnemius Muscles

**Figure 6:**
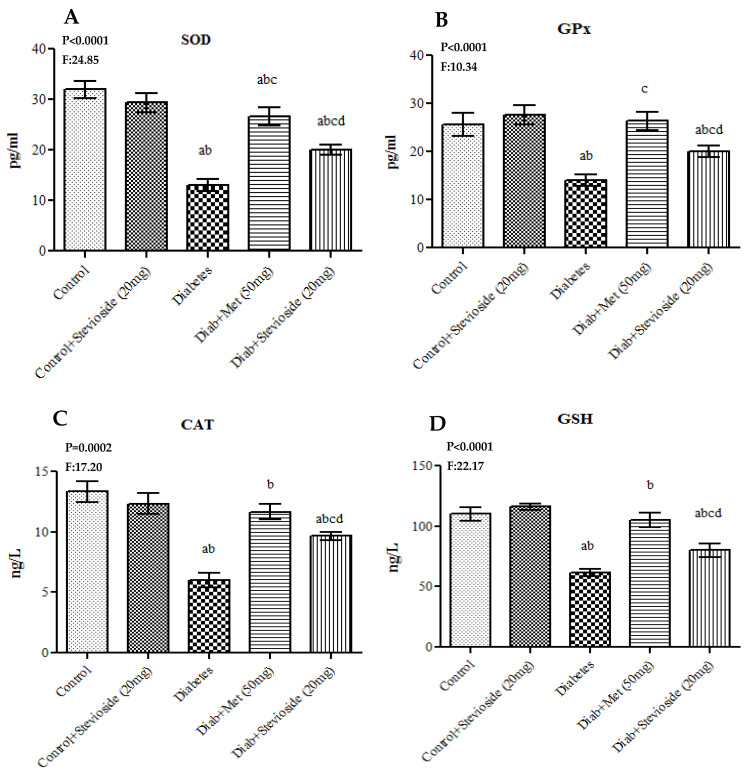
(**A**–**D**) Effect of Stevioside on antioxidant enzymes in gastrocnemius muscle of type-2 diabetic rats. Each bar represents mean ± SEM (n = 6) analyzed by one-way ANOVA with Student–Newman–Keul’s multiple comparison test. Significance at *p* < 0.05. ^a^ Significantly different from control group. ^b^ Significantly different from control rats treated with 20 mg Stevioside group. ^c^ Significantly different from diabetic group. ^d^ Significantly different from metformin treated diabetic group.

To estimate the anti-oxidative potential of Stevioside during diabetes, the levels of antioxidant enzymes including CAT, SOD, GSH and Gpx were measured in the gastrocnemius muscles of various treatment groups. The antioxidant enzymes concentration was markedly decreased in the gastrocnemius muscles of T2DM rats compared to the control rats. On the other hand, Stevioside treatment profoundly elevated the levels of these antioxidant enzymes in diabetic rats very effectively as metformin as shown in Figure 6A–D. This in turn suggests that Stevioside might exert its anti-diabetic properties by ameliorating oxidative stress in muscle tissues.

### 2.7. Stevioside Up-Regulated Muscular Gene Expression of Insulin Receptor (IR) in Type 2 Diabetic Rats

**Figure 7:**
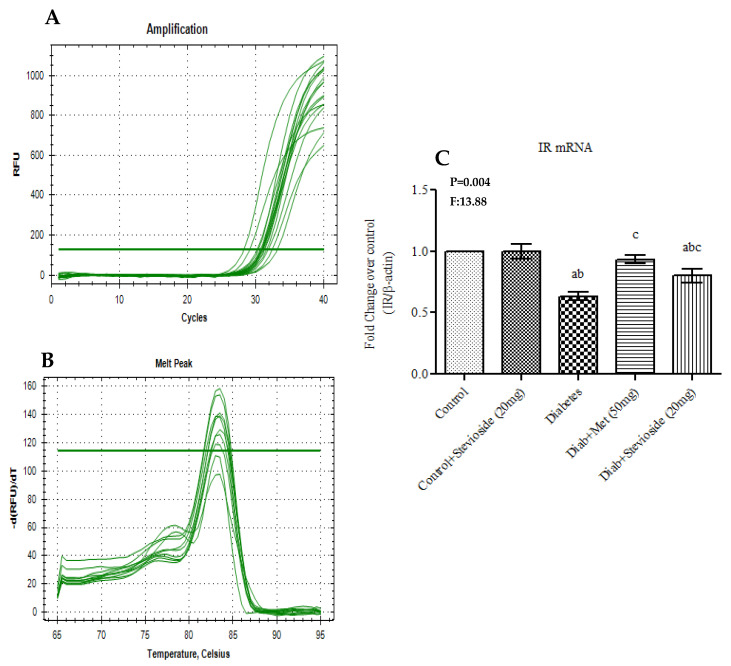
(**A**–**C**) Effect of Stevioside on insulin receptor mRNA expression in gastrocnemius muscle of type-2 diabetic rats. Each bar represents mean ± SEM (n = 6) analyzed by one-way ANOVA with Student–Newman–Keul’s multiple comparison test. Significance at *p* < 0.05. ^a^ Significantly different from control group. ^b^ Significantly different from control rats treated with 20 mg Stevioside group. ^c^ Significantly different from diabetic group. (**A**) Amplification Plot; (**B**) Melt Curve analysis; (**C**) IR mRNA expression given in fold change.

Group III T2DM rats showed a significant decrease in the gene and protein expression levels of IR compared to control rats (Figure 7A–C). However, Stevioside treatment enhanced the gene expression of IR in the gastrocnemius muscles of diabetic rats thereby suggesting its potential to enhance the insulin signaling ability of diabetic gastrocnemius muscle.

### 2.8. Stevioside Activated Akt Signaling Events in Diabetic Gastrocnemius Muscles

**Figure 8:**
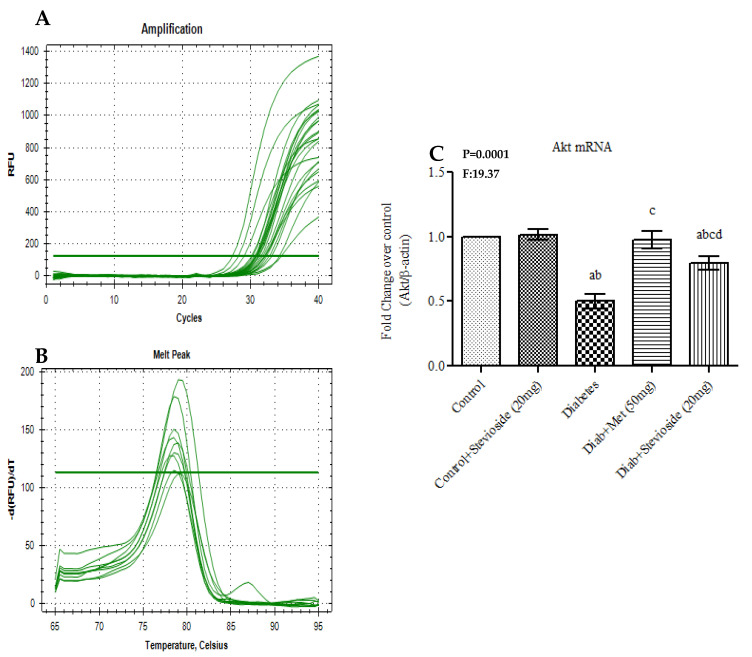
(**A**–**C**). Effect of Stevioside on Akt mRNA expression in gastrocnemius muscle of type-2 diabetic rats. Each bar represents mean ± SEM (n = 6) analyzed by one-way ANOVA with Student–Newman–Keul’s multiple comparison test. Significance at *p* < 0.05. ^a^ Significantly different from control group. ^b^ Significantly different from control rats treated with 20 mg Stevioside group. ^c^ Significantly different from diabetic group. ^d^ Significantly different from metformin treated diabetic group. (**A**) Amplification Plot; (**B**) Melt Curve analysis; (**C**) Akt mRNA expression given in fold change.

To determine Stevioside’s capacity to up-regulate Akt signaling in diabetic gastrocnemius muscles, the gene expression of Akt was evaluated. The results obtained are depicted in Figure 8A–C. In the group III diabetic rats, the mRNA level of Akt was significantly reduced suggesting disruptions in the insulin signaling pathways. The up-regulation of Akt in Stevioside treated diabetic rats suggests that Stevioside could augment Akt signaling events in insulin resistant gastrocnemius muscle.

### 2.9. Stevioside Augmented GLUT 4 Trafficking in Gastrocnemius Muscles of T2DM Rats

**Figure 9:**
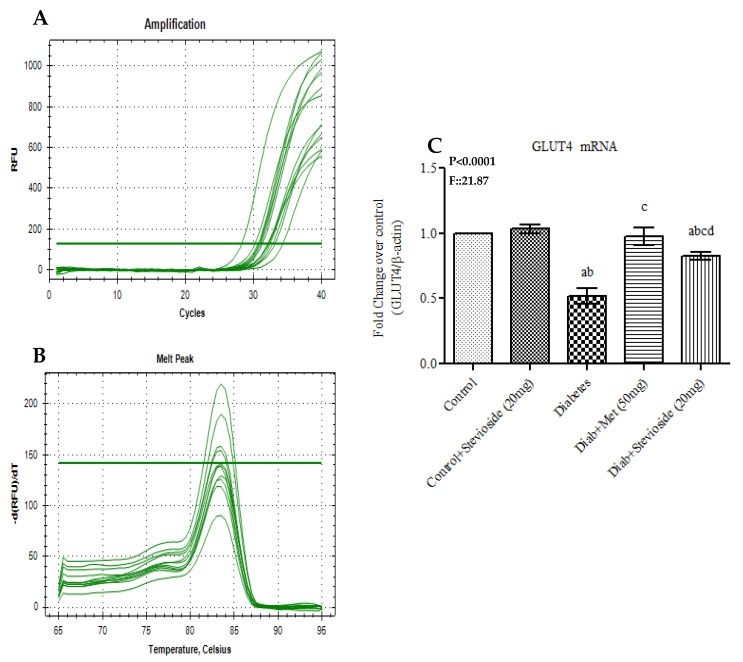
Effect of Stevioside on GLUT 4 mRNA expression in gastrocnemius muscle of type-2 diabetic rats. Each bar represents mean ± SEM (n = 6) analyzed by one-way ANOVA with Student–Newman–Keul’s multiple comparison test. Significance at *p* < 0.05. ^a^ Significantly different from control group. ^b^ Significantly different from control rats treated with 20 mg Stevioside group. ^c^ Significantly different from diabetic group. ^d^ Significantly different from metformin treated diabetic group. (**A**) Amplification Plot; (**B**) Melt Curve analysis; (**C**) GLUT4 mRNA expression given in fold change.

GLUT 4 trafficking in plasma membrane and cytosol is the key event that indicates the muscle cell’s potential to take up circulating glucose for glycolysis or glycogenesis. The gene expression level of GLUT 4 was remarkably low in gastrocnemius muscles of group III diabetic rats compared to the control group. Fascinatingly, Stevioside treatment reverses this deterrence by up-regulating GLUT 4 gene in diabetic rats as shown in Figure 9A–C. Our study results suggest that Stevioside is highly capable of promoting GLUT 4 trafficking in diabetic muscle tissues.

#### 2.9.1. Effect of Stevioside on ^14^C-2-Deoxyglucose Uptake and ^14^C-Glucose Oxidation in the Gastrocnemius Muscles

**Figure 10:**
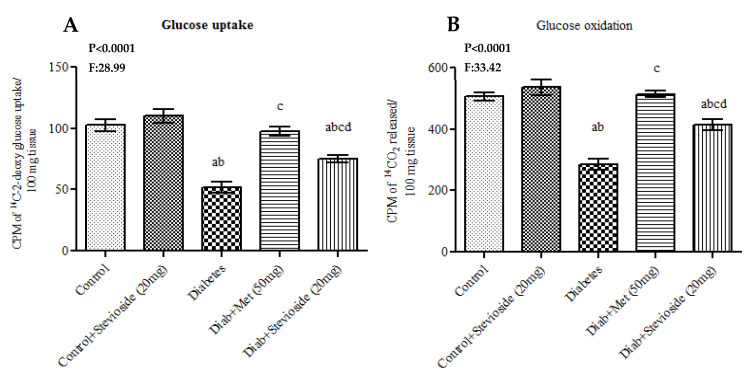
(**A**,**B**) Effect of Stevioside on GU and oxidation in gastrocnemius muscle of tpe-2 diabetic rats. Each bar represents mean ± SEM (n = 6) analyzed by one-way ANOVA with Student–Newman–Keul’s multiple comparison test. Significance at *p* < 0.05. ^a^ Significantly different from control group. ^b^ Significantly different from control rats treated with 20 mg Stevioside group. ^c^ Significantly different from diabetic group. ^d^ Significantly different from metformin treated diabetic group.

T2DM group III rats manifested poor GU and glucose oxidation potential compared to control rats. Fascinatingly, Stevioside treatment enhanced the ability of diabetic gastrocnemius muscles of group III rats to take up more glucose and also stimulated glucose oxidation very effectively like metformin (Figure 10A,B).

#### 2.9.2. Molecular Docking Studies of IRS with Stevioside

**Figure 11:**
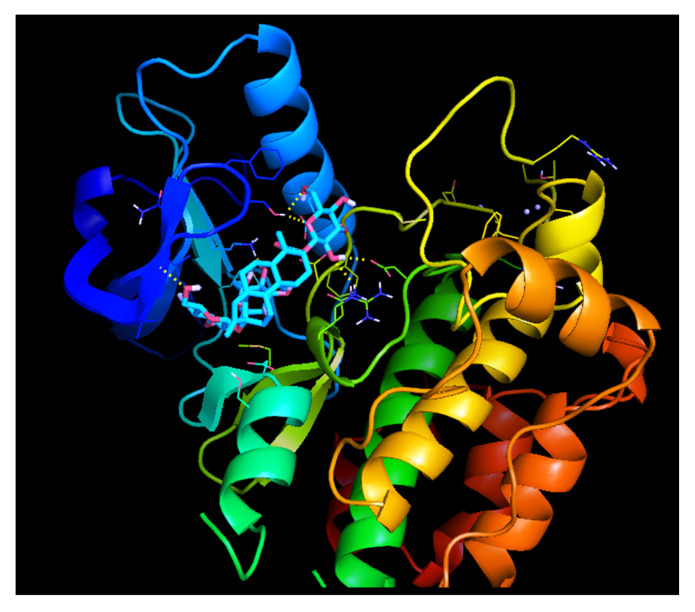
Binding interaction of Stevioside with IRS-1 Protein.

The docked position of IRS-1 with Stevioside is seen in Figure 11, which consistently indicates the binding position of the Stevioside to the IRS-1. As shown in Table 1, Stevioside with IRS-1 demonstrated higher binding energy−8.27 kcal/mol, and formed five hydrogen bond interactions with amino acids, namely GLN-977, SER-979, LYS-1003, ASP-1105 and ASN-1110 with hydrogen bond distances of 2.5 A°, 1.8 A°, 2.0 A°, 2.2 A° and 2.7 A°, respectively (Figure 11).

#### 2.9.3. Molecular Docking Studies of GLUT 4 with Stevioside

**Figure 12:**
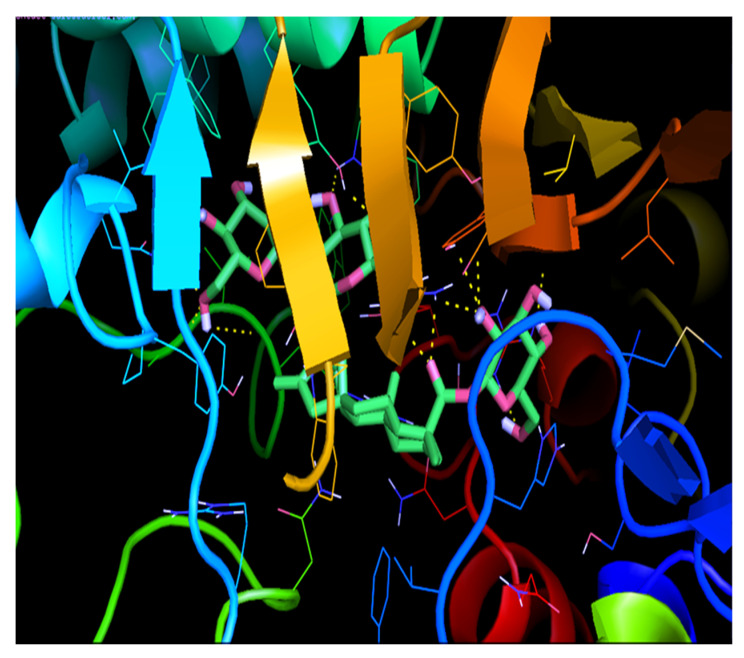
Binding interaction of Stevioside with GLUT 4 Protein.

PyRx was used for the docking study of the homology modelled protein structure of GLUT 4 which shown to be efficient in terms of the lowest docking energy as shown in Table 1 Stevioside indicated binding energy −7.8 kcal/mol having higher binding energy. In addition, Stevioside developed 10 hydrogen bonding interactions with GLUT 4 through the amino acids GLN-113, TYR-168, TRP-173, ARG-188, ALA-190, ARG-336, GLU-359 GLY-404, TYR-405 and ARG-433 and the distance of each hydrogen bond interaction as shown in Table 1 and in Figure 12.

## 3. Discussion

Skeletal muscles are the primary organs involved in the uptake of more than 60% of ingested glucose via insulin mediated signaling mechanisms [23,24,25]. Hence, resistance of skeletal muscles to insulin signals act as the key driving event for the pathogenesis of T2DM [26,27,28]. Mounting studies show that the increased oxidative stress caused by ectopic lipid accumulation and hyperglycemia trigger ROS generation and promote lipid peroxidation in muscle cell membranes which in turn impair insulin signaling, decrease GLUT 4 shuttle to PM (plasma membrane) and thereby lead to insulin resistance [29]. In this study, we investigated if Stevioside possess the potential to reverse oxidative stress mediated downregulation of insulin signaling and thus augment GLUT 4 dependent GU (glucose uptake) in gastrocnemius muscles of HFD and sucrose induced T2DM rats. As expected, Group III rats fed with HFD and sucrose showed marked increase in serum lipid profile (including FFA, TG, VLDL-c and LDL-c) and significant decrease in HDL cholesterol levels. These observations portray the development of obesity and dyslipidemia in group III rats due to excess fat intake [30]. However, Stevioside treatment in these diabetic rats significantly reduced body weight and serum lipid profile with concomitant increase in HDL cholesterol levels suggestive of its hypocholesterolemic properties. In this regard, it has been reported that administration of different doses of Stevioside to diabetic rats effectively reduced dyslipidemia [31].

Furthermore, the gastrocnemius muscles of Group III diabetic rats displayed significantly lower levels of antioxidant markers including CAT, SOD, GPx, GR and GSH and remarkably high levels of ROS including H_2_O_2_, °OH and LPO when with matched control rats. This in turn denotes the induction of oxidative stress in the muscle cells of these rats by HFD and sucrose supplementation. Hyperglycemia promotes ROS generation by increasing electron transport chain flux and subsequently promoting mitochondrial hyperpolarization. Similarly, elevated FFA increase ROS overproduction by interacting with the complex subunits of electron transport chain and thereby reducing the electron flow rate through complex I and complex III [32]. Furthermore, FFA also augment ROS synthesis from inner mitochondrial membrane by releasing cytochrome C and thereby affecting the flow of electrons from complex III to complex IV [33]. Antioxidants including CAT, SOD, GPx, GR and GSH eliminate ROS from cells without propagation by catalyzing the electronic pairing of free radicals with each other. In this study, a remarkable rise in the levels of ROS and dramatic decline in the levels of antioxidants in group III T2DM rats indicate the induction of oxidative stress caused by hyperglycemia and elevations in serum FFA levels. In diabetic gastrocnemius tissues, however, Stevioside administration considerably increased antioxidant enzyme levels, greatly lowers ROS including H_2_O_2_, °OH and avoids lipid peroxidation. Our findings are consistent with Rotimi et al. (2018) [31] wherein Stevioside scavenged the excess ROS produced by HFD, improved the antioxidants and restored the liver function of high fat fed rat model of insulin resistance [31]. Hence, our study results show that stevisode could effectively circumvent oxidative stress in the gastrocnemius muscles of type 2 diabetic rats.

Due to the induction of oxidative stress, group III diabetic rats manifest symptoms of insulin resistance which is evident by the elevated FBG and serum insulin levels accompanied by impaired glucose and insulin tolerances. Stevioside treatment significantly decreased FBG and serum insulin levels in diabetic rats and also improved their glucose and insulin tolerances as effectively as standard anti-diabetic drug, metformin. This in turn suggests that Stevioside possess the potential to treat T2DM by improving insulin sensitivity and regulating the levels of FBG. In connection with the present findings, studies have shown that oral administration of *Stevia rebaudiana* and its bioactive compounds reduced streptozotocin-induced oxidative stress mediated hyper glycemia in experimental rats [31].

The major problem underpinning cell dysfunction and T2DM development is insulin resistance in skeletal muscle [23,24]. Insulin increases GU in cells by binding to the IR present in the cell membrane and activating the downstream insulin signaling pathways under physiological conditions. Insulin stimulates receptor tyrosine kinases, which then activate IRS1 (pIRS1) by dephosphorylating serine residue 636 (Ser636) and phosphorylating tyrosine residues 632 (Tyr632). PI3K binds to pIRSTyr632 and phosphorylates Akt at serine 473 (pAkt^Ser473^) and threonine 308 to activate it (pAktThr 308). By phosphorylating AS160 at threonine 642, pAkt activates its downstream substrate AS160 (pAS160^Thr642^). Activated AS160 enhances GLUT 4 trafficking to the plasma membrane (PM), which leads to a more efficient diffusion of circulating glucose into muscle cells. In skeletal muscle cells, insulin-mediated stimulation of the IRS/PI3K/Akt/AS160 signaling cascade stimulates translocation and fusion of GLUT 4 vesicles to the PM, hence augmenting muscle GU. Skeletal muscles that are insulin resistant do not respond to insulin, which causes impairment in the insulin signaling pathway and, as a result, a reduction in GLUT 4 levels and GU by these cells [34]. As a result, we studied whether Stevioside may activate insulin signaling pathways, upregulate GLUT 4 expression and eventually improve GU in diabetic gastrocnemius muscles that are insulin resistant.

As expected, diabetic rats’ gastrocnemius muscles had considerably lower mRNA levels of IR, IRS-1, Akt and GLUT 4, indicating that oxidative stress driven activation of c-Jun NH-2 terminal kinase and extracellular signal-regulated kinases lowered IRS-1 protein activity [35]. Surprisingly, Stevioside administration increased insulin production in these rats and up-regulated the IR/IRS-1/Akt pathway in their gastrocnemius muscle by increasing the amounts of the signaling molecules. This resulted in increased GLUT 4 mRNA levels in these tissues. We also looked at whether SIT-mediated GLUT 4 upregulation could increase GU and oxidation in diabetic gastrocnemius muscles. HFD-fed rats had impaired GU and oxidation in their gastrocnemius muscles, as seen in earlier research [36], due to a lower amount of GLUT 4 as a result of an inefficient insulin signaling pathway. Surprisingly, steviosde administration, like metformin, increases GU and oxidation in diabetic gastrocnemius muscles. This could be because Stevioside has the ability to stimulate insulin signaling pathways in these tissues.

Molecular docking is a computational approach that seeks to build non-covalent binding between the protein (receptor) and the small molecule (ligand/inhibitor). Docking determines the mode of interaction between the target protein and the small ligand for the binding site. Binding energy shows the affinity of the particular ligand and potency with which the compound interacts and binds to the cavity of the target protein. A compound with lower binding energy is favored as a potential drug candidate. In order to identify the impact of Stevioside against IRS-1 and GLUT 4 proteins in diabetic molecular docking with PyRx was carried out.

PyRx was used to evaluate the effect of Stevioside compounds on IRS-1 and GLUT 4 proteins through molecular docking. The IRS-1 and GLUT 4 docking analysis with Stevioside compound showed the highest docking energy and was considered to be the strongest molecules at the target protein site (Table 1). Both the complex seems to have more than five hydrogen bond interactions and it has clearly shown that the Stevioside compound is strongly bound to the selected proteins. The distance between the H-bonds was less than three, confirming the formation of desirable interactions between ligand and receptor. Both complexes have a distance of less than three H-bonds. This also proves that the chosen compound showed better activity against IRS-1 and GLUT 4 protein targets.

## 4. Materials and Methods

### 4.1. Chemicals

Stevioside was procured from ChemCruz™ biochemicals. ^14^C-2-deoxyglucose and ^14^C-glucose were obtained from Board of Radiation and Isotope Technology, India; Lipid profile assay kit was procured from Spinreact (Girona, Spain). Gene specific primers including IR, IRS-1, Akt and GLUT 4 and β-actin primers were obtained from Eurofins Genomics India Pvt Ltd., Bangalore, India. Basic reagents and chemicals needed for the study were procured from MP Biomedicals, LLC, 9 Goddard, Irvine, CA-92618, United States of Americ (USA), Sisco Research Laboratories, India and Sigma Aldrich, USA.

### 4.2. Animal

Healthy 5–6 months old male Wistar rats (180 ± 20 g) were uninterruptedly supplemented with pelleted diet and clean water ad libitum during the study and animal maintenance was done according to the National Guidelines. The animal study protocols were approved by the Institutional Animal Ethics committee (IAEC No: BRULAC/SDCH/SIMATS/IAEC/02-2019/022, accessed on 25 February 2019).

### 4.3. Induction of T2DM

T2DM was induced in the rats under study by the administration of HFD which is composed of 66% standard rat feed, 30% coconut oil, 3% cholesterol and 1% cholic acid for a period of 60 days. In addition to HFD, the rats were also fed with 30% sucrose via drinking water. To ascertain the induction of diabetes, rats were allowed to fast overnight on the 58th day of the experimental period and FBG levels were measured. Rats with FBG level >120 mg/dL were regarded as T2DM rats. HFD and sucrose water were administered to the rats till the end of the study.

### 4.4. Experimental Design

Rats were randomly categorized into four groups (n = six rats per group). Group I-Control; Group II-Control rats orally administered with Stevioside (20 mg/kg/day) for 45 days; Group III-T2DM rats; Group IV-T2DM rats orally administered with the conventional anti-diabetic drug metformin (50 mg/kg/day) for 45 days. Group V-T2DM rats orally administered with Stevioside (20 mg/kg/day) for 45 days and Oral Glucose Tolerance test (OGTT) and Fasting Blood Glucose (FBG) were done in all the animals two days before sacrifice. After the experimental period, animals were sacrificed, blood was collected, serum was separated and stored at −80 °C. Gastrocnemius muscle was excised immediately from the rats and stored at −80 °C until further analysis.

### 4.5. FBG Measurement

In the animals fasted overnight, blood was collected from the tip of rat tail and the FBG levels were measured using On-Call Plus blood glucose test strips (ACON Laboratories Inc., 10125 Mesa Rim Road San Diego, CA 92121, USA). Results were expressed mg/dL.

### 4.6. Oral Glucose Tolerance Test (OGTT)

After overnight fasting, FBG levels were measured in the animals under study. Then, glucose load (10 mL/kg; 50% *w*/*v*) was given orally to the rats and the blood glucose levels were measured at three different time points (60, 120 and 180 min) using strip blood glucose test strips. The FBG value was considered as a zero-minute value. Results were expressed mg/dL.

### 4.7. Liver and Renal Function Markers

Biochemical-assay kits from Spin react, Spain, were used to detect liver function markers such as alanine transaminase (ALT), aspartate transaminase (AST) and alkaline phosphatase (AST), as well as kidney function markers (urea and creatinine). The results were expressed as U/L.

### 4.8. Estimation of Fasting Serum Insulin

Fasting serum insulin levels were measured using ultrasensitive rat insulin ELISA kit (Crystal Chem Inc., Elk Grove Village, IL, USA) according to manufacturer’s protocol. Insulin concentration was expressed as mU/L.

### 4.9. Serum Lipid Profile Analysis

Serum triglyceride (TG), free fatty acid (FFA), total cholesterol (TC), high-density lipoproteins (HDL) and low-density lipoproteins (LDL) were estimated colorimetrically using Spinreact assay kit (Spain) as per manufacturer’s protocol. Results were expressed as mg/dL.

#### 4.9.1. Estimation of ROS Levels in Gastrocnemius Muscles

The levels of oxidative stress markers including LPO, H_2_O_2_ and OH° in the gastrocnemius muscles of rats under study were measured spectrometrically using the methods previously described [37,38,39]. Results were expressed in nmol/L and U/L respectively.

#### 4.9.2. Measurement of Antioxidant Enzymes in Gastrocnemius Muscles

Antioxidant markers including catalase (CAT), superoxide dismutase (SOD), Glutathione (GSH) and Glutathione peroxidase (Gpx) were estimated in the gastrocnemius muscles of rats under study using the methods previously described [40,41,42,43].

#### 4.9.3. Glucose Uptake by Gastrocnemius Muscles

GU in gastrocnemius muscles was estimated using ^14^C-2-deoxyglucose in a liquid scintillation counter by the methods previously described [44]. Briefly, gastrocnemius tissue samples cut into 100 mg pieces were first incubated in Krebs–Ringer bicarbonate (KRB) buffer containing 8 mM glucose at 37 °C for 60 min in 95% air—5% CO_2_ and then in KRB buffer supplemented either with insulin (to estimate insulin mediated GU) or without insulin (to estimate basal GU) at 37 °C for 20 min and finally incubated in KRB buffer supplemented with ^14^C-2-deoxyglucose (0.05 µCi) for 20 min at 37 °C in 95% air—5% CO_2_. The difference in the radioactivity between the samples incubated with and without insulin was measured in liquid scintillation counter and the results were expressed as CPM of ^14^C-2-deoxyglucose uptake/100 mg tissue.

#### 4.9.4. Glucose Oxidation in Gastrocnemius Muscles

Glucose oxidation in gastrocnemius muscles was estimated using ^14^C-glucose by the methods previously described [45]. Briefly, 100 mg tissue was placed in a container that contains 170 µL DMEM medium, 10 IU penicillin and 0.05 µCi ^14^C-glucose and the container was supplied with 95 % air and 5 % CO_2_ for 30 s. The containers were then closed tightly using a rubber lid with CO_2_ trap and incubated at 37 °C. The CO_2_ trap was changed once every two hours. After the removal of second trap, further metabolism and residual CO_2_ release from the samples were halted by adding 1 mL of 1N H_2_SO_4_. The containers were again locked airtight for one hour before the removal of third and final trap. Then, the radioactivity of all the CO_2_ traps was measured in a Beta counter and the results are expressed as CPM of ^14^CO_2_ released/100 mg tissue.

#### 4.9.5. mRNA Expression Analysis

##### Total RNA Isolation, cDNA Conversion and Real-Time PCR

Using a TRIR kit (Total RNA Isolation Reagent Invitrogen), total RNA was isolated from control and experimental samples. In brief, to 100 mg of fresh muscle tissue, 1 mL of TRIR was added and homogenized [46,47]. The content was transferred to a microcentrifuge tube instantly and 0.2 mL of chloroform was added, vortexed for 1 min then kept at 4 °C for 5 min. Later, the contents were centrifuged at 12,000× *g* for 15 min at 4 °C. The aqueous phase (upper layer) was carefully transferred to a fresh microfuge tube and an equal volume of isopropanol was added, vortexed for 15 s and placed on ice for 10 min. After centrifugation of the content at 12,000× *g* for 10 min at 4 °C, the supernatant was discarded and RNA pellet was washed with 1 mL of 75% ethanol by the vortex. The isolated RNA was estimated spectrometrically and the RNA concentration was expressed in microgram (μg).

By using the reverse transcriptase kit from Eurogentec (Seraing, Belgium), complementary DNA (cDNA) was synthesized from 2 μg of total RNA as stated in the manufacturer’s protocol. To perform real-time PCR [46,48], the reaction mixture containing 2× reaction buffer (Takara SyBr green master mix), Forward and reverse primers of the target gene and house-keeping gene, water and β-actin (the primer sequences were listed in Table 2) in total volume of 45 μL expect the cDNA was made, mixed intensively and spun down. In individual PCR vials, about 5 μL of control DNA for positive control, 5 μL of water for negative control and 5 μL of template cDNA for samples were taken and reaction mixture (45 μL) was added. Forty cycles (95 °C for 5 min, 95 °C for 5 s, 60 °C for 20 s and 72 °C for 40 s) was set up for the reaction. Data analysis was performed by the 2^−ΔΔCt^ method using CFX 96 Real Time system software (Bio-Rad) with β-actin as reference gene.

#### 4.9.6. Statistical Analysis

Data analysis was carried out using Graph Pad Prism version 5 software. The significance in the variation of each parameter between four different groups was determined by one-way analysis of variance (ANOVA) with Student–Newman–Keul’s multiple comparison test as post hoc. Results were presented as mean + SEM. In Duncan’s test, *p* < 0.05 was considered to be statistically significant.

#### 4.9.7. In Silico Analysis

##### Protein Preparation

There are two proteins included in docking analysis, one of which is an insulin receptor substrate-1 (IRS-1). The 3D dimensional structure of the IRS-1 protein (PDB ID; 1K3A) was accessed from the Brookhaven Protein Data Bank (PDB). The second is the GLUT 4 protein. Since the crystal structure of the protein GLUT 4 was not available in the PDB database, the three-dimensional structure of GLUT4 was modelled using a homology modelling approach.

##### Homology Modelling of GLUT4 Protein

The homology modelling of the GLUT4 protein was conducted using the software MODELLER 9v9.19. The human GLUT4 amino acid sequence was collected from the Swiss-Prot protein sequence database. The accession number of the protein is P14672 and the sequence contains 509 amino acids. The first step involved searching a number of similar sequences to select a template for finding a related protein of GLUT4. In order to classify the homologous proteins from the Protein Data Bank (PDB), the GLUT4 protein was subjected to a PSI-BLAST search through the NCBI server (http://www.ncbi.nim.nih.gov/blast, accessed on 15 October 2021). The PDB ID 4PYP was chosen as the model template as it has an identity score of 65 percent. Homology modelling was performed for the GLUT4 protein against the chosen 4PYP template using the MODELLER 9v9.19 program. The results of the modelled systems were graded as per their internal score function. The modelled structure with the lowest internal scores was defined and analyzed for Model Validation.

##### Protein Preparation

The input file (target receptor protein) was created by removing water molecules, ions, ligands and sub-units from the original structure file. Kollman charges and polar hydrogen atoms were applied to the PDB receptor file for the preparation of the receptor protein for docking simulation.

##### Ligand Preparation

The chemical composition of stevioside was extracted from the PubChem compound database. It was prepared with the ChemBioDraw and the MOL SDF format of this ligand was translated to a PDBQT file using the PyRx method to produce atomic coordinates [53].

##### Molecular Docking

The computational ligand-target docking technique was used to evaluate the structural complexes of IRS-1 and GLUT4 (targets) with stevioside (ligand) in terms of understanding the structural basis of this target protein specificity. Molecular docking was performed by PyRx, the Auto Dock Vina option depending on the score feature. “Grid point” is allocated to the interaction energy of Stevioside with IRS-1 and GLUT4. At each stage of the process, the interaction energy of ligand and protein was measured using grid computed atomic affinity potentials [54,55,56].

## 5. Conclusions

Our present findings clearly show that Stevioside attenuates high fat diet-induced insulin resistance and improves glycemic control in diabetic gastrocnemius muscle by facilitating the expression of insulin signaling molecules and GLUT4. To the best of our knowledge, the present findings are the first to report a possible role of Stevioside on insulin signaling molecules in high fat and sucrose-induced insulin resistant model. Hence, our study concludes that supplementation of Stevioside can provide a constructive approach for the management of type-2 diabetes. Further studies on identifying the role Sativoside on human cell line model are warranted in order to ascertain its mechanism of action towards clinical trials engaging such natural drug to treat T2DM.

## Figures and Tables

**Table 1:** Molecular docking results of Stevioside with GLUT 4 and IRS-1.

Protein Name	Binding Affinity (Kcal/mol)	H-Bond Details	Distance
GLUT 4	−7.8	GLN-113	2.3
		TYR-168	2.5
		TRP-173	2.2
		ARG-188	2.3
		ALA-190	2.3
		ARG-336	2.6
		GLU-359	2.1
		GLY-404	2.2
		TYR-405	2.6
		ARG-433	2.0
IRS-1	−8.2	GLN-977	2.5
		SER-979	1.8
		LYS-1003	2.0
		ASP-1105	2.2
		ASN-1110	2.7

**Table 2:** List of primers used.

Name of the Gene	Primer Sequence	Reference
Rat IR	Sense primer: 5′-GCC ATC CCG AAA GCG AAG ATC-3′	[49]
	Anti-sense primer: 5′-TCT GGG TCC TGA TTG CAT-3′	
Rat Akt	Sense primer: 5′-GGA AGC CTT CAG TTT GGA TCC CAA-3′	[50]
	Anti-sense primer: 5′-AGT GGA AAT CCA GTT CCG AGC TTG-3′	
Rat GLUT 4	Sense primer: 5′-GGG CTG TGA GTG AGT GCT TTC-3′	[51]
	Anti-sense primer: 5′-CAG CGA GGC AAG GCT AGA-3′	
Rat β-actin	Sense primer: 5′-AAG TCC CTC ACC CTC CCA AAA G-3′	[52]
	Anti-sense primer: 5′-AAG CAA TGC TGT CAC CTT CCC-3′	

## Data Availability

The data presented in this study are available in this article.

## Sample Availability

Samples of the compounds are not available from the authors.
